# Efficacy of topical tenofovir against transmission of a tenofovir-resistant SHIV in macaques

**DOI:** 10.1186/s12977-015-0195-z

**Published:** 2015-08-08

**Authors:** Charles W Dobard, Sunita Sharma, Mian-er Cong, Rolieria West, Natalia Makarova, Angela Holder, Chou-Pong Pau, Debra L. Hanson, Francis J Novembre, Jose Gerardo Garcia-Lerma, Walid Heneine

**Affiliations:** Laboratory Branch, Division of HIV/AIDS Prevention, Centers for Disease Control and Prevention, MS G45, 1600 Clifton Road, Atlanta, GA 30329 USA; Quantitative Sciences and Data Management Branch, Division of HIV/AIDS Prevention, Centers for Disease Control and Prevention, Atlanta, GA USA; Yerkes Primate Center, Emory University, Atlanta, GA USA

**Keywords:** Pre-exposure prophylaxis, HIV-1 drug resistance, Tenofovir, Microbicides

## Abstract

**Background:**

Topically delivered tenofovir (TFV) from intravaginal rings, tablets, or gels is being evaluated for HIV prevention. We previously demonstrated that TFV delivered vaginally by gel protected macaques from vaginal infection with SHIV. Here we investigated efficacy of the TFV gel against vaginal transmission of a TFV-resistant SHIV containing the K65R mutation (SHIV162P3_K65R_) and its relationship to drug levels in vaginal tissues.

**Results:**

SHIV162P3_K65R_ shows approximately a 5-fold reduction in susceptibility to TFV compared to wild-type SHIV. Efficacy was evaluated in pig-tailed macaques exposed vaginally twice-weekly (up to 10 weeks) to SHIV162P3_K65R_ 30 min after receiving placebo (n = 6) or 1% TFV (n = 6) gel. Four of the six controls were infected after a median of 5 exposures. In contrast, five of six macaques that received TFV gel remained uninfected after 20 vaginal SHIV162P3_K65R_ exposures, resulting in an estimated efficacy of 75%. The mean intracellular TFV-diphosphate (TFV-DP) concentrations in vaginal lymphocytes 4 h after a single gel dose were found to be high (1,631 fmol/10^6^ cells, range 492–3,847) and within the in vitro IC_75_ range (1,206 fmol/10^6^ cells) for SHIV162P3_K65R_.

**Conclusion:**

Both the modest resistance conferred by K65R and the high TFV-DP exposure in vaginal lymphocytes, likely explain the observed protection. The findings in this model do not predict complete loss of protection by topical TFV against vaginal exposure to HIV-1_K65R_ viruses and provide a tissue drug target for high efficacy. These data will facilitate the development of TFV delivery platforms that have high activity on both wild-type and TFV-resistant viruses.

## Background

As treatment programs for human immunodeficiency virus (HIV) expand, access to antiretroviral therapies continue to benefit persons infected with HIV, particularly in Sub-Saharan Africa, the epicenter of the pandemic [1]. Research on HIV prevention through the use of oral or topical antiretroviral drugs has also accelerated to provide promising prevention options for uninfected persons to protect themselves from acquiring HIV. Oral emtricitabine (FTC) and tenofovir (TFV) disoproxil fumarate (TDF) administered daily has now been demonstrated to effectively prevent HIV in men and women [2] and is now an approved HIV prevention medication in the USA. Likewise, various topical products that dose vaginal tissues with tenofovir are under development. These include vaginal gels or tablets, as well as intravaginal rings formulated with TFV or its oral pro-drug TDF. A phase IIb clinical trial in South Africa (CAPRISA 004) demonstrated that a vaginal 1% TFV gel reduced acquisition of HIV by an average of 39% and approximately 54% in women who used the gel more than 80% of the time [3]. However, recent data released from a similar phase III trial in South Africa (FACTS 001) was unable to show efficacy by TFV gel, likely due to low adherence by study participants [4]. While sub-analysis of this study demonstrated an ~55% protective effect amongst women identified as highly adherent, it is unclear if these data will support regulatory approval of this TFV-releasing product [4]. Implications of these results however, may include advancing the clinical development of other TFV-releasing topical products such as vaginal tablets or intravaginal rings that share the high biological efficacy of TFV gels but may be more desirable by women [5–7]. Previous pharmacokinetic studies have shown that 1% TFV gels, vaginal TFV tablets containing 10 mg of TFV, and intravaginal rings with either TFV or TDF all dose vaginal tissues with high concentrations of TFV-diphosphate, the pharmacologically active drug [6–8]. Furthermore, efficacy studies in macaques exposed vaginally to wild-type SHIV demonstrated high protection by both 1% TFV gel and TDF intravaginal rings [7–9].

As in treatment, the use of topical TFV for prevention raises drug resistance concerns for its potential to impact the efficacy of antiretroviral prophylaxis as TFV is a main component of first-line regimens used globally to treat persons infected with HIV [1]. A K65R mutation in the reverse transcriptase gene of HIV-1, which confers low-level (~2–5 fold) resistance to TFV, has been documented in patients failing TFV-containing regimens and may increase exposure of uninfected persons to TFV-resistant HIV-1_K65R_ viruses. The K65R mutation is also observed in persons virologically failing while on stavudine (d4T)-containing therapy, a first-line regimen extensively implemented in early treatment programs in the developing world [10, 11]. Clinical studies can help define the impact of TFV-resistant virus on the efficacy of topical TFV products. However, prevention failures due to infection with a TFV-resistant virus are difficult to ascertain in humans because of the uncertainty on adherence to product at the time of infection and the inability to exclude acquired resistance post-infection, which requires early testing and is often not feasible. Furthermore, the K65R mutation is known to have a high fitness cost to viral replication, and thus is often rapidly outgrown by the more fit wild-type virus [12–14]. The reversion of K65R can be facilitated by insufficient drug pressure through inconsistent product use and low systemic TFV exposures, which could underestimate clinical K65R-related TFV failures.

Animal models conducted under well-controlled conditions including consistent product dosing and challenge viruses with defined drug resistance profiles, can be utilized for assessing the impact of TFV-resistant viruses on the efficacy of topical TFV. The repeat low-dose (RLD) SHIV exposure macaque model is an established model of vaginal and rectal HIV transmission which predicted the prophylactic efficacy of oral FTC/TDF combination [15–18]. This model was further used to evaluate the efficacy of FTC/TDF against drug-resistant SHIV containing either K65R or the emtricitabine-associated M184V mutation [13, 19]. Likewise, the vaginal transmission model demonstrated complete protection against wild-type SHIV by vaginal 1% TFV gel when applied 30 min before challenge and ~74% protection when administered 3 days prior to challenge [8, 9]. By linking efficacy to tissue drug levels following gel dosing, this model was the first to document the intracellular drug concentrations required for vaginal protection [8]. Analysis of TFV-diphosphate (TFV-DP) in vaginal lymphocytes, pointed to TFV-DP concentrations above the 95% inhibitory concentration (IC_95_) as a good predictor for protection. This pharmacologic correlate of protection was further validated by the demonstration of high efficacy of TDF intravaginal rings against vaginal SHIV infection [7]. Here, we expand the use of the macaque model to evaluate the efficacy of topically delivered TFV from a gel against a TFV-resistant SHIV containing the K65R mutation that recapitulates the resistance and fitness profile of HIV-1 with the K65R mutation.

## Results

### Efficacy of TFV gel against SHIV162P3_K65R_

**Fig. 1:**
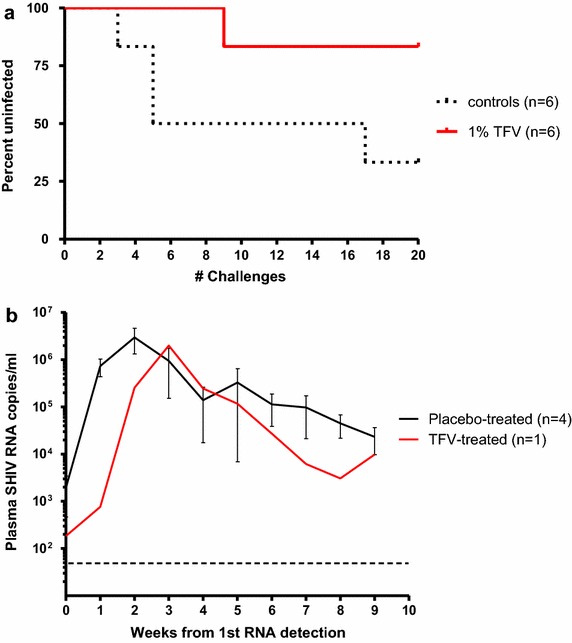
Efficacy of TFV gel against SHIV162P3_K65R_. **a** Survival curves representing the cumulative percentage of uninfected macaques as a function of the number of challenges. After 20 SHIV162P3_K65R_ exposures, challenges were stopped and animals were monitored for ten additional weeks for infection in the follow-up period. **b** Breakthrough infections show no evidence of blunted viremia. Individual virus load kinetics of controls (*black lines*) and breakthrough infection (*red line*) under continued twice-weekly gel dosing. Time zero indicates the time of first SHIV RNA detection in plasma. Time zero indicates the time of first SHIV RNA detection in plasma. The *dashed line* denotes the limit of quantification of the virus load assay (50 copies/ml).

Vaginal 1% TFV gel provided complete protection against wild-type SHIV162P3 when applied 30 min before virus challenge [9]. Figure 1 shows infection outcomes after a maximum of 20 challenges in macaques who received either placebo or 1% TFV gel 30 min before vaginal exposure to SHIV162P3_K65R_. Four of six macaques in the placebo group became infected after a median of 5 challenges (at exposure 3, 5, 5, and 17). In contrast, five of six macaques in the TFV group remained SHIV negative as measured by both PCR and serology throughout the challenge period and 10-week washout. The breakthrough infection in the TFV treatment group occurred at challenge 8. The efficacy of TFV gel was estimated at 75% based upon 4/6 infections in the control group compared to 1/6 in the TFV treated group. However, due to small group numbers and low transmissibility of SHIV162P3_K65R_ (67% infection rate), the protective effect in animals treated with TFV gel and risk of infection between the two groups was not statistically significantly (p = 0.24, Fisher’s exact test).

Analysis of SHIV reverse transcriptase sequences at time of infection confirmed the presence of K65R in all infected animals (data not shown). The SHIV-infected breakthrough animal continued to receive 1% TFV gel treatment for up to 10 weeks after the first RNA detection. Figure 1b shows the kinetics of plasma viral replication in the breakthrough infection compared to the 4 controls. The peak viremia in the breakthrough infection (2.9 × 10^6^ vRNA copies/ml) was within the range seen in placebo controls (3.5 × 10^5^ to 1.7 × 10^7^ vRNA copies/ml), as was the area under the curve (5.4 × 10^6^ and 2.7 × 10^6^ copies/ml) over the first 10 weeks of infection (*p* = 0.39), showing no difference in systemic viremia during acute infection.

### Systemic drug absorption following vaginal dosing with 1% TFV gel

**Fig. 2:**
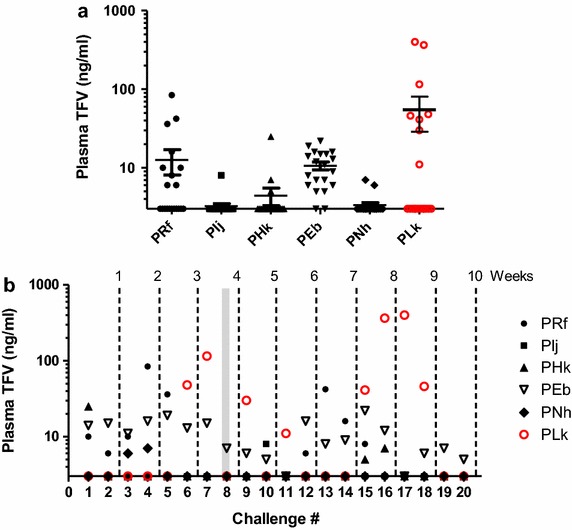
Systemic drug exposure following vaginal TFV gel dosing. **a** Cumulative plasma TFV levels in macaques following twice-weekly dosing over the 10 week challenge period. **b** Longitudinal assessment of individual plasma TFV levels in macaques at time of each SHIV challenge (30 min post gel dosing). TFV concentrations (LLOD = 3 ng/ml) are shown in *black* and *red* for uninfected and infected macaques, respectively. *Shaded gray bar* indicates estimated time of infection.

In line with earlier reports, overall plasma TFV levels detected 30 min after vaginal TFV gel application (12.2 ng/ml; range = 0–400 ng/ml) were low in all six TFV treated macaques (Fig. 2a) [8, 9]. Likewise, the proportion of measureable TFV in plasma over the 10 week challenge period was relatively low, with only 41 of a total of 120 measurements (34%) above the limit of detection (Fig. 2b). Both the low systemic exposure and infrequent detection of TFV likely reflects the temporal changes in vaginal drug absorption associated with the menstrual cycle [8, 20, 21]. Interestingly, the mean TFV concentrations in the breakthrough infection macaque PLk (52 ng/ml) was higher than the levels seen in uninfected animals (4.6 ng/ml). However, we note that the TFV exposures in this macaque at and prior to the estimated time of infection (challenge 8) was within the range of the protected animals, and thus the continued twice-weekly TFV dosing post-infection greatly contributed to the overall high TFV levels detected in this animal (Fig. 2b).

### Intracellular TFV-DP concentration in vaginal lymphocytes predict efficacy against TFV-resistant SHIV

We previously documented in this model a strong association between intracellular TFV-DP levels in vaginal lymphocytes that exceeded the in vitro IC_95_ and protection in vivo, suggesting that TFV-DP levels in target cells is a good predictor of high efficacy against wild-type SHIV transmission. Similar analysis were performed to determine if the relationship between efficacy and tissue drug levels following TFV dosing also applies to protection against SHIV162P3_K65R_. We assessed the intracellular TFV-DP concentrations in vaginal lymphocytes purified from vaginal tissues 4 h after dosing macaques with 1% TFV gel and compared them to concentrations achieved in lymphocytes dosed in vitro at TFV concentrations corresponding to the inhibitory concentrations (IC_50–95_) for SHIV162P3_K65R_. Data from seven macaques (4 current and 3 historical from a previous study [8]) showed that the median TFV-DP concentration in vaginal lymphocytes at 4 h was 1,631 fmol/10^6^ cells (range = 492–3,847 fmol/10^6^ cells). In vitro dosing of PBMCs at TFV concentrations in range of the IC_50_ (12 µM), IC_75_ (60 µM), and IC_95_ (300 µM) values for SHIV162P3_K65R_ resulted in mean (±std dev) intracellular TFV-DP levels of 320 ± 141, 1,206 ± 235 and 4,559 ± 2,867 fmol/10^6^ cells, respectively. These data show that in vivo TFV-DP levels were closest to the IC_75_ concentrations which is consistent with the observed 75% protection found in this study. These data are in agreement with previous findings against wild-type SHIV162P3 and provide drug target levels needed for protection against SHIV162P3_K65R_.

## Discussion

HIV treatment programs with regimens containing either TDF or d4T have the potential to enrich for TFV-resistant HIV-1 containing the K65R mutation among persons experiencing virologic failure on these regimen, thus raising questions about efficacy of topical TFV products for prevention among uninfected persons exposed to such viruses [6, 7, 11]. Here, we show in a macaque model that TFV delivered by vaginal gel maintained protection against a SHIV isolate with the K65R mutation that shares the same TFV resistance and replicative fitness profile with HIV-1_K65R_. We provide pharmacokinetic evidence in support of efficacy by showing inhibitory intracellular TFV-DP concentrations in vaginal lymphocytes within the in vitro IC_75_. Both the modest 5-fold resistance to TFV conferred by K65R and the high levels of TFV-DP in vaginal lymphocytes likely explain the observed protection in this model. These findings suggest other topical delivery platforms including intravaginal rings, tablets, or films that are capable of delivering TFV concentrations that exceed those achieved by vaginal gels will equally protect against both wild-type and TFV-resistant viruses.

We demonstrate that five of six macaques treated with vaginal TFV gel were uninfected compared to only two of six placebo gel controls. However, the inability to infect two controls together with the small group numbers (n = 6), resulted in insufficient power to draw statistically significant conclusions. The low in vivo transmissibility of SHIV162P3_K65R_ at virus doses that efficiently infect macaques with wild-type SHIV162P3 is consistent with the high fitness cost conferred by K65R and lower transmission rate observed previously [12, 14]. The virus infectivity to virion particle ratio of SHIV162P3_K65R_ was found to be ~17-fold lower than that of wild-type SHIV162P3, and increasing the challenge dose ten times in this study was helpful but not sufficient to achieve 100% infection rate in the placebo group [8, 9]. Although K65R has been previously shown to rapidly revert to wild-type post-infection, we document persistence of K65R in all infected animals, likely the result of introducing two mutations (AAA) in the K65 codon versus the single nucleotide mutation often found in naturally emerging K65R mutations. These findings further support the use of this K65R isolate as a tool for executing TFV resistant transmission and prevention studies under well-controlled conditions [12].

We further showed that TFV was detected in plasma samples collected shortly after gel dosing, confirming rapid absorption through vaginal tissues. The low and infrequent systemic exposure of TFV in macaques following twice-weekly intravaginal dosing were similar to those detected in women who applied vaginal TFV gel twice-daily for 2 weeks (5.5 ng/ml) [22]. We also found no reduction in the acute viremia of the breakthrough infection compared to controls, reflecting insignificant antiviral activity due to nominal systemic TFV exposure by the continued twice-weekly gel dosing post infection.

The single-dose PK data were instrumental in linking TFV-DP concentrations in vaginal lymphocytes to in vitro activity and in vivo protection against SHIV162P3_K65R_. It is plausible that the tissue concentrations may have slightly increased during the challenge period due to accumulation of TFV-DP by repeat twice-weekly gel dosing, underscoring the need to achieve high mucosal tissue levels to fully protect against TFV-resistant viruses. As interest increases in exploring alternative delivery platforms capable of administering TFV prodrugs that achieve even higher vaginal tissue concentrations of TFV-DP because of sustained release or more efficient dosing, it is expected that such products may further enhance protection against TFV-resistant virus [5–7, 23]. The protective tissue drug levels defined in this study will help inform these studies. We also note that while our results address the efficacy against low-level TFV resistance mediated by K65R, our findings may have broader implications on mutant viruses with a similar level of TFV resistance conferred by other mutations as seen clinically with multiple thymidine analog mutations.

## Conclusions

We show in a repeat challenge model that 1% TFV gel maintained protection against vaginal infection with a TFV-resistant SHIV and provide pharmacokinetic support for this protection. These findings are reassuring, particularly for regions with prevalent HIV-1 with the K65R mutation. This macaque model of pharmacokinetics and efficacy against vaginal SHIV162P3_K65R_ infection will help inform the development of improved topical TFV products that may have higher and more durable efficacy against TFV-resistant isolates and are more desirable and easier to adhere to by women.

## Methods

### Gel formulation

Tenofovir [(R)-9-(2-phosphonylmethoxypropyl)adenine] (TFV) was kindly provided by Gilead Sciences; 1% TFV (wt/wt) was formulated in 2% hydroxyethyl cellulose (HEC) gel as previously described [8, 9]. Gels were formulated at pH 6.5 to mimic the average vaginal pH of pigtailed macaques [21]. A matching placebo 2% HEC gel was used as a control.

### Virus stocks

The wild-type SHIV162P3 and SHIV162P3_K65R_ virus stocks used were generated as described elsewhere [14]. The K65R substitution in SHIV162P3 confers ~5-fold reduction in susceptibility to TFV and was introduced by site-directed mutagenesis with two nucleotide changes (AAA → CGA) to minimize reversion of K65R in vivo [13]. Virus titer of challenge stocks was calculated in macaque PBMCs and diluted to 500 tissue culture infective dose (500 TCID_50_) and stored separately in 1-ml aliquots in liquid nitrogen. Individual vials were thawed on ice prior to each challenge.

### Measurement of drug concentrations in plasma, vaginal lymphocytes, and peripheral blood mononuclear cells (PBMCs)

TFV concentrations in plasma were measured in macaques 30 min after vaginal administration of TFV gel, resulting in the analysis of 20 samples from each of the 6 macaques (120 total). Briefly, TFV was extracted from 100 µl of plasma by protein precipitation with 500 µl of methanol containing 200 ng of 13C-labeled TFV as internal standard. Supernatant containing the drug from precipitation was evaporated to near dryness under vacuum and then re-suspended in HPLC buffer containing 9.9 mM of acetic acid, 5.9 mM of ammonium hydroxide, and 9.4 mM of formic acid (pH ~3). Drug levels were analyzed by using liquid chromatography–mass spectrometry (LC–MS) [8, 24]. The assay had a lower limit of quantification (LLOQ) of 3 ng/ml and standard curve R^2^ values greater than 0.99.

We previously documented high intracellular TFV-DP concentrations in lymphocytes collected from vaginal tissues in macaques (n = 3) sacrificed 4 h following a single dose of vaginal 1% TFV gel [8]. To expand these data, we additionally measured TFV-DP levels in vaginal lymphocytes in SHIV-infected macaques (n = 4) sacrificed 4 h after receiving a single vaginal dose of 1% TFV gel. All tissue collection and processing procedures were conducted by the same veterinarian pathologists, laboratory technicians, and using the same tissue digestion and mononuclear cell enrichment protocols as described in the previous study [8]. Briefly, vaginal tissue collected at time of necropsy was dissociated using enzyme cocktails and lymphocyte purification procedures. Total cell populations were gated and counted for mononuclear cells using a Muse Cell counter with CytoSoft Data Acquisition and Analysis Software (Millipore, Billerica, MA). Intracellular TFV-DP concentrations in vaginal lymphocytes were measured with an automated online weak anion exchange solid-phase extraction method coupled with ion-pair chromatography–MS/MS [24]. TFV-DP levels were expressed as femtomoles (fmol) per million cells with a lower limit of quantitation (LLOQ) of 2.5 fmol/sample.

To compare TFV-DP concentrations observed in vivo with those achieved in vitro, macaque PBMCs were incubated with varying concentrations of TFV and intracellular TFV-DP concentrations were measured at each dose. Briefly, PBMCs (5.0 × 10^6^) were incubated 2–4 h in RPMI media containing TFV concentrations within the range of the 10–99% inhibitory concentrations (IC_10–99_) [300, 60, 12, 2.4, 0.48, 0.096 µM] for wild-type and SHIV162P3_K65R_ virus [8, 13]. Following incubation, cells were washed extensively with saline buffer solution, pelleted, and lysed in 1 ml of ice cold 80% MeOH. Intracellular TFV-DP levels were measured as described above.

### Efficacy of 1% TFV gel in preventing vaginal transmission of SHIV162P3_K65R_

The efficacy of TFV gel against vaginal transmission of SHIV162P3_K65R_ was evaluated in female pig-tailed macaques under conditions similar to those described for wild-type SHIV162P3 [8, 9]. Macaques received 3 ml of intravaginal placebo (n = 6) or 1% TFV (n = 6) gel 30 min before each vaginal exposure to SHIV162P3_K65R_. Challenges were performed twice per week (every 3–4 days) for 10 weeks or up to 20 exposures. Vaginal challenges were administered by atraumatic inoculation of 1 ml of SHIV162P3_K65R_ (500 TCID) into the vaginal vault. The challenge dose was increased ~10 times higher than wild type SHIV162P3 (from 50 to 500 TCID_50_) to adjust for the lower transmissibility SHIV162P3_K65R_ [8, 9, 12, 14]. Blood was collected 30 min after each gel application to monitor for SHIV infection and plasma drug levels. SHIV infection was determined by monitoring SHIV RNA in plasma by RT-PCR [8]. The estimated time of infection was defined as 7 days (two challenges) prior to SHIV positive to account for the eclipse period between virus inoculation and detection of SHIV RNA in plasma [25]. Animals were considered protected if they tested negative for SHIV plasma RNA and SHIV DNA in PBMCs and remained seronegative during the course of the study and the following 10 weeks of washout in the absence of challenge and gel application. All experiments were done under highly controlled conditions by the same personnel, using the same virus stock, and procedures as described in previous studies [8, 9, 20]. These studies adhered to the Guide for the Care and Use of Laboratory Animals (Institute for Laboratory Animal Research, 1996); all procedures were approved by the Institutional Animal Care and Use Committees (IACUC) of both the Centers for Disease Control and Prevention (CDC) and the Yerkes National Primate Research Center (Emory University).

### Statistical analysis

The cumulative probability of macaques remaining uninfected after repeated low-dose viral exposures was computed and graphically displayed using the product limit (Kaplan–Meier) estimator. The log-rank test statistic was used to non-parametrically compare survival curves between the control and treatment groups. Uninfected macaques were right censored at the maximal exposure number (20 exposures). Intervention efficacy was calculated as 1 − (p_1_/p_0_), where p_1_ and p_0_ denote the proportion of infections for intervention and control animals, respectively. Acute RNA viremias were compared using the Wilcoxon rank-sum test.
